# A multifunctional hydrogel with hemostatic and photothermal properties for burn repair†

**DOI:** 10.1039/d5ra03968a

**Published:** 2025-07-14

**Authors:** Chunbiao Wu, Hao Jiang, Qiang Zhang, Ying Jiang, Yi Bao, Shulin Fan, Juelan Ye, Hao Wang, Jianru Xiao

**Affiliations:** a Institute of Orthopedic Biomedical and Device Innovation, School of Health Science and Engineering, University of Shanghai for Science and Technology No. 580 Jungong Road Shanghai 200093 China Juelan_Ye@163.com wang_hao@usst.edu.cn xiaojr83@163.com; b Department of Orthopedic Oncology, Shanghai Changzheng Hospital, Naval Medical University Shanghai 200003 China; c Department of Plastic Surgery, The Affiliated Hospital of Yangzhou University, Yangzhou University Hanjiang Middle Road Yangzhou 225009 China; d Department of Urology, The Third Affiliated Hospital of Naval Medical University No. 700 Moyu North Road, Jiading District Shanghai 201805 China; e Department of Clinical Medicine, Clinic Medical College of Anhui Medical University Wangjiang West Road Anhui 230031 China

## Abstract

Effective management of burn wounds requires multifunctional dressings with integrated hemostatic, antibacterial, antioxidant, and regenerative properties. Herein, we developed an injectable hydrogel by grafting dopamine onto γ-polyglutamic acid (γ-PGA), followed by crosslinking with carboxymethyl chitosan (CMCS) *via* EDC/NHS chemistry. The resulting PGDA/CMCS hydrogel exhibited excellent injectability, bioadhesion, and mechanical stability (compressive modulus: 1.15 kPa), along with high swelling capacity (up to 1179%) and controlled degradation. The hydrogel showed potent ROS-scavenging activity (78.2% for DPPH˙), efficient photothermal conversion (31.05%), and enhanced antibacterial effects under NIR irradiation. *In vitro* assays confirmed good cytocompatibility (>90% viability) and hemocompatibility (hemolysis <5%). *In vivo*, the hydrogel rapidly stopped bleeding in tail and liver injury models and significantly promoted burn wound healing, especially when combined with photothermal stimulation. This multifunctional hydrogel holds strong potential for clinical use in hemorrhagic and infected wound management.

## Abstract 2

A multifunctional hydrogel composed of PGDA and CMCS was developed *via* amide and hydrogen bonding. The hydrogel exhibits strong adhesion, antioxidant capacity, and rapid hemostasis, accelerating burn wound healing under NIR-enhanced conditions.

## Graphical abstract

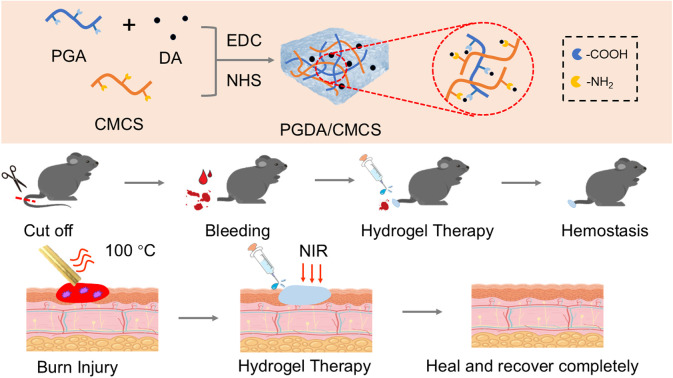

## 1. Introduction

Burn injuries pose a major threat to human health and safety, with statistical data indicating that approximately 7 to 12 million people worldwide suffer burns annually, resulting in around 200 000 deaths.^1^ Non-lethal burns can require prolonged hospitalization and lead to disfigurement, disability, and psychosocial burdens.^2^ Among these, third-degree burns result in extensive damage to both the epidermal and dermal tissues, leading to inflammation, fluid loss, and compromised barrier function, which significantly elevates the risk of infection and complicates wound healing.^3,4^

Autologous skin grafting remains the standard treatment, but limitations such as donor site scarcity and immune rejection restrict its utility.^5,6^ Advanced wound dressings, especially hydrogels, offer unique benefits like maintaining a moist environment, promoting hemostasis, and protecting against infection.^7–10^ However, many existing hydrogel dressings have single functions or insufficient mechanical strength.^11,12^ In designing an ideal dressing for burn management, key requirements include: (1) efficient exudate absorption; (2) moisture retention to promote faster healing; (3) robust antibacterial properties; and (4) excellent biocompatibility.

Polyglutamic acid (γ-PGA) is a natural, biodegradable polymer primarily derived from *Bacillus subtilis*.^13^ With glutamic acid units connected by amide bonds, γ-PGA features high water solubility and outstanding biocompatibility.^14^ One of the most notable characteristics of γ-PGA is its exceptional water absorption capacity, making it an ideal candidate for applications requiring liquid retention and high moisturization, particularly in burn wound dressings.^15,16^ The high-water retention capacity of γ-PGA hydrogels helps maintain a moist wound environment, which is essential for hemostasis and wound healing. It keeps wounds hydrated, promotes cell migration and proliferation, and reduces pain and infection risk.^17^ Additionally, the high biocompatibility and biodegradability of γ-PGA make it an ideal choice for hydrogel wound dressings.

In this work, we aim to address these challenges by designing a γ-PGA-based hydrogel grafted with dopamine and crosslinked with CMCS, leveraging the catechol chemistry and amide crosslinking strategy to realize a stable, multifunctional network. We hypothesize that the synergistic effects of dopamine-mediated adhesion, γ-PGA's moisture regulation, and CMCS's bioactivity will endow the hydrogel with strong hemostatic efficiency, antioxidant capability, and enhanced wound healing potential, particularly in severe burn environments.

**Scheme 1:**
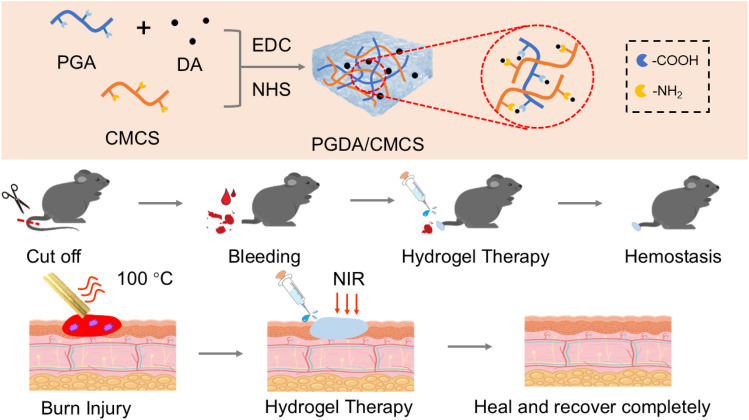
Preparation of PGDA/CMCS hydrogel for hemostasis and burn wound repair. The PGDA/CMCS hydrogel was fabricated using CMCS and PGA as the primary structural components, followed by chemical crosslinking with EDC/NHS. The resulting hydrogel demonstrated effective hemostatic properties and significantly promoted wound healing in a severe burn model.

In response to the complex clinical demands of burn wound dressings, a multifunctional hydrogel was constructed with integrated capabilities including wet adhesion, antibacterial efficacy, antioxidation, and enhanced wound healing. γ-PGA was grafted with dopamine (DA) and crosslinked with carboxymethyl chitosan (CMCS) *via* amide bonds to form an interpenetrating PGDA/CMCS hydrogel network (Scheme 1). The hydrogel is easily prepared using EDC/NHS activation, enabling covalent bonding with tissue proteins and enhancing dopamine-mediated adhesion to moist surfaces. The incorporation of γ-PGA helps create a locally dry environment, enhancing adhesion, promoting platelet aggregation, and absorbing exudates for effective hemostasis. Meanwhile, the antioxidant properties of the PGDA/CMCS hydrogel reduce inflammation, support cell proliferation and migration, and accelerate collagen deposition, collectively promoting wound healing. These results indicate strong clinical potential for PGDA/CMCS hydrogel in rapid hemostasis and burn wound repair.

## 2. Experimental methods

### 2.1 Materials

γ-Polyglutamic acid (γ-PGA, *M*_w_ > 700 000 Da), dopamine hydrochloride (DA), *N*-hydroxysuccinimide (NHS), 1-ethyl-3-(3-dimethylaminopropyl) carbodiimide hydrochloride (EDC·HCl), and carboxymethyl chitosan (CMCS, 90–150 kDa, degree of substitution 80%) were purchased from Shanghai Macklin Biochemical Co., Ltd (Shanghai, China). Calcein-AM/PI Live/Dead Cell Double Staining Kit was obtained from Solarbio Life Sciences (Beijing, China). 4% paraformaldehyde solution was supplied by Biosharp Life Sciences (Hefei, China). Trypsin-EDTA solution was purchased from LABGIC Technology Co., Ltd (Beijing, China). The Cell Counting Kit-8 (CCK-8) was obtained from MedChemExpress LLC (Shanghai, China). Fetal bovine serum (FBS), penicillin–streptomycin solution (PS, Hyclone®), and Dulbecco's Modified Eagle Medium (DMEM) were acquired from Thermo Fisher Scientific (Waltham, MA, USA). *Escherichia coli* (ATCC 25922) and *Staphylococcus aureus* (ATCC 25923) were provided by the Ministry of Health Inspection Center (China). The L929 fibroblast cell line was purchased from Wuhan Pricella Biotechnology Co., Ltd (Wuhan, China). All reagents were of analytical grade and used as received without further purification. Deionized (DI) water was used throughout all experiments.

### 2.2 Preparation of PGDA

To synthesize dopamine-grafted PGA (PGDA), 1 g of γ-PGA was dissolved in 100 mL deionized water under stirring, followed by addition of 0.25 g DA, 0.5 g NHS, and 1 g EDC at room temperature for 12 h. The reaction mixture was placed in a 14 kDa dialysis bag for 5 days, then frozen at −20 °C overnight and lyophilized to yield PGDA.

### 2.3 Preparation of PGDA/CMCS hydrogel

To prepare the hydrogel precursor solutions, PGDA and CMCS were separately dissolved in deionized water to obtain concentrations of 2% (w/v) and 4% (w/v), respectively. Prior to gelation, a defined amount of EDC (0.4 mmol) and NHS (0.2 mmol) was added to 300 μL of PGDA solution to activate 0.1 mmol of carboxyl groups, calculated based on the degree of dopamine substitution. After activation for 10 minutes at room temperature, 100 μL of CMCS solution was added to the PGDA solution, and the mixture was stirred gently to allow crosslinking between carboxyl groups on PGDA and amino groups on CMCS. The resulting hydrogel was designated as PGDA/CMCS. For comparison, a PGA/CMCS hydrogel was prepared using the same procedure, except that ungrafted PGA was used instead of PGDA.

### 2.4 Characterization of hydrogel

The chemical structure of the hydrogels was analyzed using Fourier-transform infrared (FT-IR) spectroscopy (Nicolet iS10, Thermo Fisher Scientific, USA) in the wavenumber range of 4000–400 cm^−1^ to confirm the formation of amide bonds and successful incorporation of dopamine. The microstructure of the lyophilized hydrogels was observed by scanning electron microscopy (SEM, SU8010, Hitachi, Japan). For SEM analysis, hydrogel samples were frozen at −20 °C, freeze-dried overnight, cut into cross-sections, and sputter-coated with gold prior to imaging.

### 2.5 Swelling and degradation properties of hydrogel

Lyophilized PGA/CMCS and PGDA/CMCS hydrogels were immersed in PBS at 37 °C. Samples were periodically removed and weighed to determine the swelling ratio. Swelling curves were plotted from these measurements. Weigh the hydrogels and record the data, plotting the swelling kinetics curve and calculating the swelling ratio using the formula:
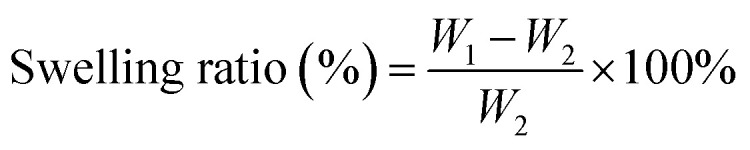
where *W*_1_ represents the weight of the swollen hydrogel, and *W*_2_ denotes the initial weight of the hydrogel.

Degradation was assessed by incubating hydrogels in PBS for up to 14 days. Lyophilizing at each time point and measuring remaining mass yielded degradation curves. The degradation ratio of the hydrogels is expressed as the remaining hydrogel mass:
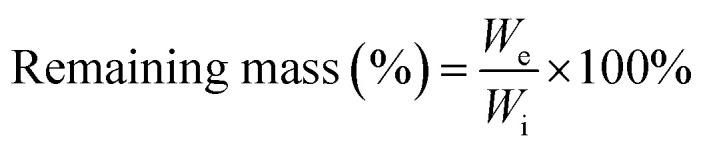
where *W*_i_ is the initial mass of the hydrogel, and *W*_e_ is the mass of the hydrogel after degradation.

### 2.6 Mechanical performance evaluation

Rheological tests were performed on mixed precursor solutions (PGA/CMCS or PGDA/CMCS) at 37 °C using a rotational rheometer (MCR 302, Anton Paar, Austria, 1 Hz, 1 mm gap). The sol–gel transition was noted when *G*′ surpassed *G*′′.

The compressive mechanical properties of the hydrogels were tested using a universal testing machine (Instron 5943, Instron Corp., USA). Compressive strength was measured by molding each hydrogel into cylinders (10 mm diameter) and compressing them at 1 mm min^−1^ until 80% strain. The compressive modulus was derived from the linear portion of the stress–strain curve.

### 2.7 Evaluation of photothermal conversion performance

PGDA/CMCS hydrogels (50–200 mg mL^−1^) were placed in small containers with 200 μL PBS and irradiated by an 808 nm NIR laser (2 W cm^−2^) for 5 min. Additional trials varied laser power (0.5–2.5 W cm^−2^) or repeated on/off cycles for photothermal stability. The photothermal conversion efficiency (*η*) was then determined using the following equation:
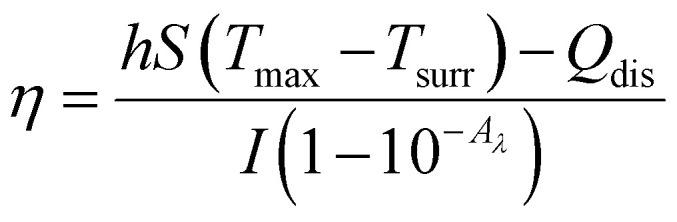
where *h* is the heat transfer coefficient, *S* is the surface area of the PGDA/CMCS hydrogel exposed to near-infrared radiation, *T*_max_ is the maximum temperature of the hydrogel, *T*_surr_ is the ambient temperature during the test, *I* is the near-infrared power, *A*_*λ*_ is the absorbance of the hydrogel at 808 nm, and *Q*_dis_ represents the heat generated by PBS under near-infrared irradiation. Additionally, *hS* can be determined using the following formula:
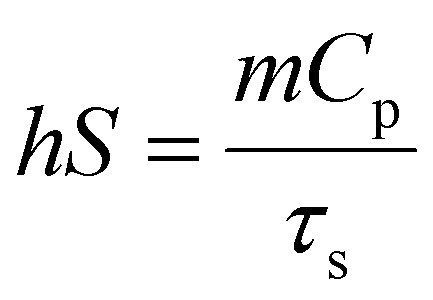
where *m* and *C*_p_ denote the mass and heat capacity of PBS, respectively. The value of *τ*_s_ is extracted from the time *vs.* −ln *θ* curve during the PGDA/CMCS hydrogel cooling phase. This curve is obtained using the following formula:
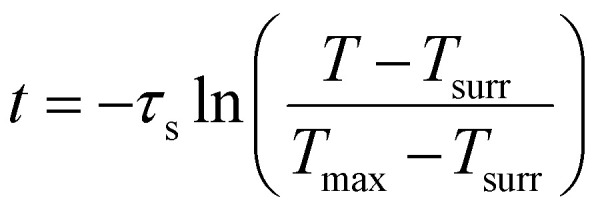
where *T* represents the real-time temperature of the PGDA/CMCS hydrogel at time *t* during the cooling process.

### 2.8 *In vitro* antioxidant assessment

Radical scavenging was measured using DPPH˙, ABTS˙^+^, and PTIO˙ assays. Hydrogel extracts at various concentrations were incubated with each radical solution for designated times, and absorbance was recorded. The DPPH˙, ABTS˙^+^, and PTIO˙ scavenging ratio was calculated based on absorbance changes using the following formula:
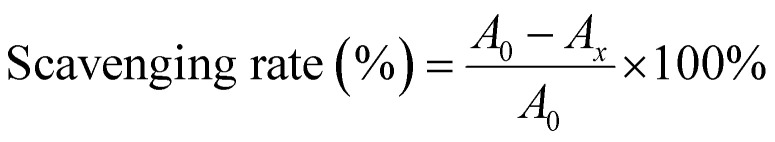
where *A* and *A*_0_ are the absorbance values of the DPPH˙, ABTS˙^+^, and PTIO˙ solution with and without PGDA/CMCS hydrogel, respectively.

### 2.9 *In vitro* antibacterial evaluation

Agar plating was conducted to evaluate antibacterial activity. After treatment, bacterial suspensions were serially diluted and plated on LB agar (Luria-Bertani, HopeBio, China). Plates were incubated at 37 °C for 24 h, and the number of colony-forming units (CFUs) was counted manually. For photothermal treatment, NIR laser (0.8 W cm^−2^, 5 min) was applied prior to incubation. Afterward, colony counts were determined by plating diluted suspensions.

### 2.10 *In vitro* biocompatibility evaluation

#### 2.10.1 Cell viability test

Cell viability (L929) was measured by culturing cells with hydrogel extracts (10–100 mg mL^−1^) for 24 h, using CCK-8 and live/dead staining.

#### 2.10.2 Hemocompatibility test

Hemocompatibility was assessed by incubating mouse red blood cells (RBC) with hydrogel and measuring hemolysis at 540 nm. The hemolysis ratio was calculated using the following equation:
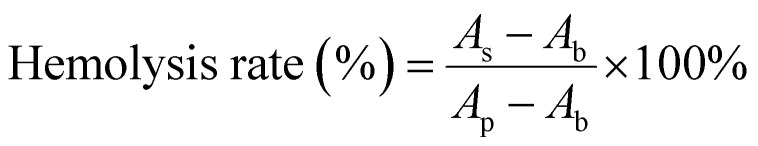
where *A*_s_*A*_b_ and *A*_p_ represent the absorbance of the sample, negative control, and positive control, respectively.

### 2.11 Evaluation of hemostatic performance in animal models

#### 2.11.1 Tail hemorrhage model

All animal procedures were approved by the Animal Ethics Committee of Yangzhou University (approval no. 202503316). Hemostatic efficacy was evaluated in SD rats using tail hemorrhage and liver injury models. For the tail model, cylindrical hydrogel samples (20 mm diameter) of PGA/CMCS and PGDA/CMCS were prepared. Rats were randomly assigned to three groups: (1) blank control, (2) Surgiflo™ (positive control), and (3) PGDA/CMCS hydrogel. After 24 h acclimation, rats were anesthetized with chloral hydrate, and one-third of the tail was amputated. Bleeding was allowed to flow onto filter paper; hydrogels or Surgiflo™ were applied as appropriate. After 3 min, filter papers were weighed to determine blood loss.

#### 2.11.2 Liver injury model

To assess internal hemostasis, rats were randomly divided into the same three groups. After anesthesia with tribromoethanol, the liver was exposed *via* laparotomy, and a standardized incision was made. Hemostatic agents were applied, and time to hemostasis was recorded. Blood loss was calculated based on the difference in body weight before and after treatment.

### 2.12 Assessment of wound healing in severe burn models

The wound healing efficacy of PGDA/CMCS hydrogels was assessed using a full-thickness burn model in C57 mice (6–8 weeks, 20–30 g, *n* = 20). After one week of acclimation, mice were anesthetized with 5% tribromoethanol (50 mg kg^−1^, i.p.), and dorsal hair was removed. A 12 mm full-thickness burn was induced using a heated copper rod applied for 40 s. Mice were randomly divided into four groups: (1) control, (2) PBS, (3) PGA/CMCS hydrogel, and (4) PGDA/CMCS hydrogel + NIR.

Treatments were administered immediately after injury and continued for 14 days. Wound images were captured on days 1, 4, 7, 10, and 14, and wound area was analyzed using ImageJ to calculate closure rates:
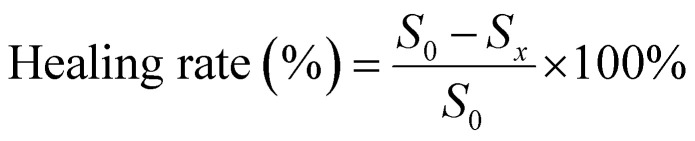
where *S*_*x*_ is the wound area at different time points, and *S*_0_ is the initial wound area.

### 2.13 Histological analysis

On day 14, wound tissues and major organs were harvested, fixed, embedded, and sectioned. H&E staining revealed epithelialization, collagen deposition, and inflammatory cell infiltration. Organ sections (heart, liver, spleen, lungs, kidneys) were examined for systemic toxicity.

### 2.14 Statistical analysis

Data are presented as mean ± standard deviation (SD). Each experimental group was repeated at least three times. Statistical analysis was performed using analysis of variance (ANOVA), followed by Scheffé's post hoc test. A *p*-value < 0.05 was considered statistically significant.

## 3. Results and discussion

### 3.1 Characterization of PGDA/CMCS hydrogel

**Fig. 1:**
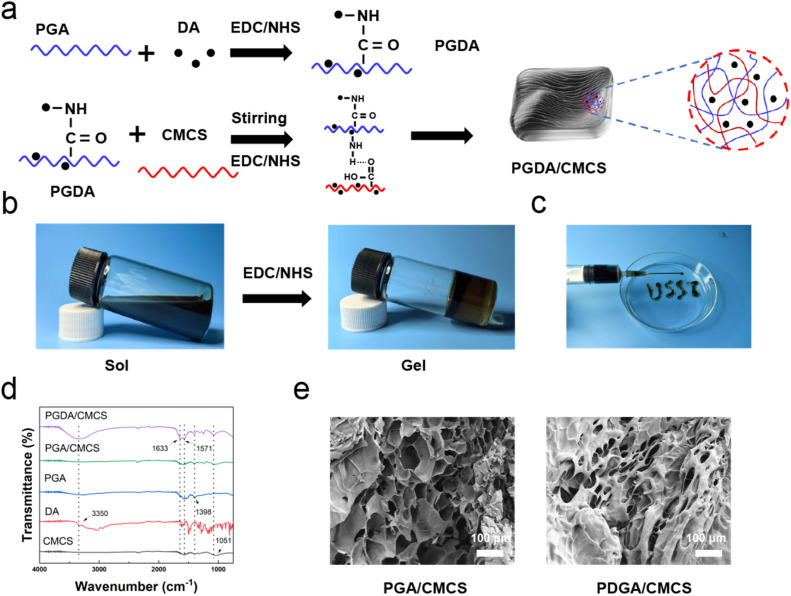
(a) Schematic illustration of the synthesis process of PGDA/CMCS hydrogel. (b) Photographs illustrating the solution-to-hydrogel transition of PGDA/CMCS hydrogel. (c) Custom pattern drawn using PGDA/CMCS hydrogel (syringe: 10 mL; needle: 22 gauge). (d) FT-IR spectra of PGDA/CMCS, PGA/CMCS, PGA, DA, and CMCS hydrogels. (e) SEM images of PGDA/CMCS and PGA/CMCS hydrogels.

As illustrated in Fig. 1a, PGDA was synthesized *via* the classic carbodiimide coupling reaction (EDC/NHS method).^18^ During this process, the carboxyl groups on PGA were activated to form reactive esters, which subsequently reacted with DA. The reaction was carried out in the acidic medium maintained by PGA, which inhibits dopamine self-polymerization and favors covalent grafting. Simultaneously, the phenolic hydroxyl groups in DA formed hydrogen bonds with the carboxymethyl groups of CMCS molecules (Fig. S1†). As shown in Fig. 1b, the combination of DA and CMCS alone, even after 6 hours of reaction, failed to produce a hydrogel. However, under the catalytic effect of EDC/NHS, the activated carboxyl groups of γ-PGA reacted with the amino groups of CMCS to form amide bonds, leading to the transformation of the solution into hydrogel. The PGDA/CMCS hydrogel exhibited excellent injectability, as demonstrated in Fig. 1c, where the hydrogel was successfully used to write the letters “USST”. This behavior can be attributed to the physical crosslinked network formed *via* electrostatic interactions and hydrogen bonds between PGA and CMCS, which is easily disrupted under low shear forces to facilitate flow.^19^ Following injection, the hydrogel swiftly regained its three-dimensional architecture, with the dopamine-mediated viscoelasticity contributing to enhanced structural integrity and formability. Infrared spectroscopy (Fig. 1d) confirmed the formation of amide bonds, with characteristic stretching vibrations observed at 1650 cm^−1^ (carbonyl groups in amide bonds) and 3350 cm^−1^ (amino groups). SEM imaging (Fig. 1e) revealed the porous structure of both PGA/CMCS and PGDA/CMCS hydrogels, with PGDA/CMCS exhibiting a denser porous architecture. This was attributed to the covalent interactions between the amine groups in DA and the carboxyl groups in PGA and CMCS, which increased crosslinking density and reduced pore size.

### 3.2 Mechanical properties of PGDA/CMCS hydrogel

**Fig. 2:**
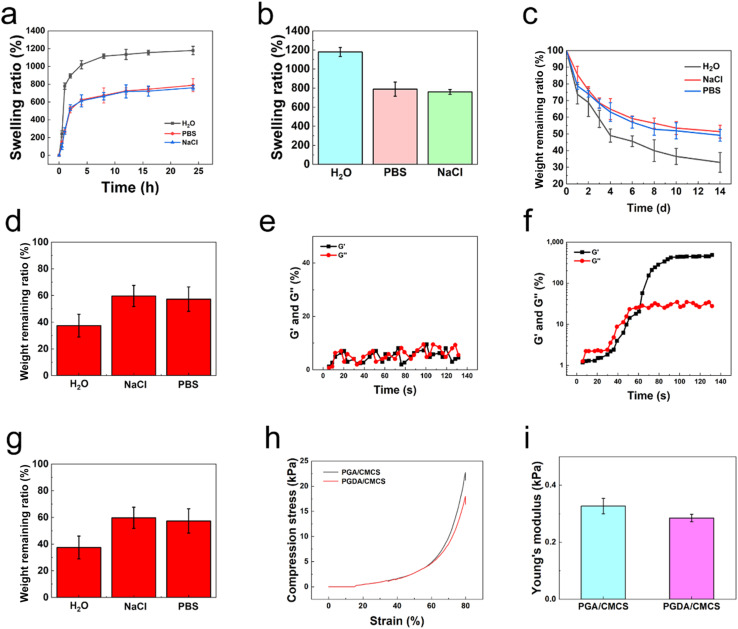
(a) Swelling curves of PGDA/CMCS in water, PBS, and saline. (b) Swelling ratios of PGDA/CMCS in water, PBS, and saline. (c) Degradation curves of PGDA/CMCS in water, PBS, and saline. (d) Degradation ratios of PGDA/CMCS in water, PBS, and saline. (e) Rheological curves of PGDA and CMCS solution without EDC/NHS. (f) Rheological curves of PGDA and CMCS solution with EDC/NHS. (g) Rheological curves of PDA and CMCS solution with EDC/NHS. (h) Compression curves of PGDA/CMCS and PGA/CMCS hydrogels. (i) Young's modulus for PGDA/CMCS and PGA/CMCS hydrogels.

The swelling properties of PGA/CMCS and PGDA/CMCS hydrogels were analyzed at 37 °C in deionized water, PBS, and saline for 24 hours (Fig. 2a). Swelling behavior followed three distinct phases: (1) rapid initial swelling due to hydrophilic surface groups and water-retentive pores, (2) slower intermediate swelling governed by diffusion ratios, and (3) equilibrium swelling with no further change. PGDA/CMCS hydrogels achieved swelling ratios of 1179%, 789%, and 760% in deionized water, PBS, and saline, respectively (Fig. 2b). The high swelling ratio in water was attributed to the hydrophilic components in the hydrogel, while lower ratios in PBS and saline were due to their higher osmotic pressures.^20^ Furthermore, the carboxyl groups in γ-PGA formed hydrogen bonds with water, enhancing water absorption and swelling.^21^

The degradation of hydrogels was evaluated in water, PBS, and saline. Amide bond hydrolysis led to faster degradation in water due to higher water molecule activity and stronger solvation capabilities, compared to PBS and saline, which contained ions that reduced solvation (Fig. 2c and d).^22^ Rheological analysis confirmed gel formation (Fig. 2e). Initially, the loss modulus (*G*′′) was higher than the storage modulus (*G*′), indicating the absence of crosslinking and a liquid-like state. After introducing EDC/NHS, *G*′ increased, intersecting *G*′′ about 60 s, and continued to rise, indicating hydrogel formation. Adding DA extended gelation time, suggesting its polymerization-modulating effect.^23^

Compression testing (Fig. 2h and i) revealed that PGDA/CMCS hydrogels exhibited higher compressive strength and Young's modulus (0.327 kPa) compared to PGA/CMCS hydrogels (0.285 kPa). This improvement was attributed to the formation of polydopamine (PDA) as a secondary crosslinker, enhancing network stability and mechanical strength. PDA's adhesive properties further reinforced hydrogel matrix interactions, resulting in greater structural stability.^24^

### 3.3 ROS scavenging properties of PGDA/CMCS hydrogel

Wound infection and the accumulation of reactive oxygen species (ROS) during the healing process can lead to oxidative stress, exacerbating inflammation and delaying recovery.^25^ To evaluate the radical scavenging ability of PGDA/CMCS hydrogels, three model radicals were selected: nitrogen-centered radicals ABTS˙^+^ and DPPH˙, and oxygen-centered radical PTIO˙. The PGDA/CMCS hydrogel, enriched with catechol groups, demonstrated a high concentration-dependent and time-dependent radical scavenging capability due to the presence of reductive hydroxyl groups.

**Fig. 3:**
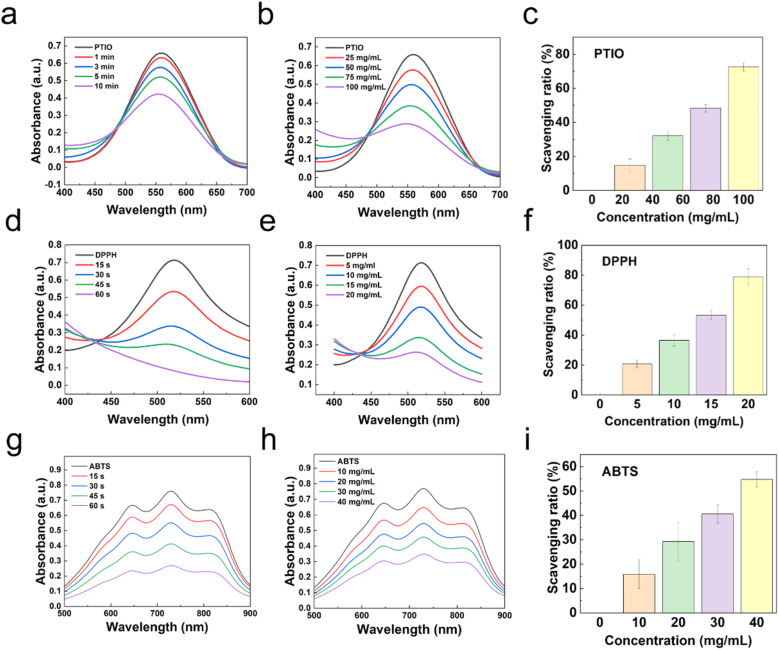
(a) UV spectrum of PGDA/CMCS hydrogel scavenging PTIO at different times. (b) UV spectrum of PGDA/CMCS hydrogel scavenging PTIO at different concentrations. (c) Scavenging ratio of PTIO by PGDA/CMCS hydrogel at different concentrations within 5 min. (d) UV spectrum of PGDA/CMCS hydrogel scavenging DPPH at different times. (e) UV spectrum of PGDA/CMCS hydrogel scavenging DPPH at different concentrations. (f) Scavenging ratio of DPPH by PGDA/CMCS hydrogel at different concentrations within 30 s. (g) UV spectrum of PGDA/CMCS hydrogel scavenging ABTS at different times. (h) UV spectrum of PGDA/CMCS hydrogel scavenging ABTS at different concentrations. (i) Scavenging ratio of ABTS by PGDA/CMCS hydrogel at different concentrations within 30 s.

For PTIO˙ radicals, the hydrogel exhibited concentration- and time-dependent scavenging behaviors (Fig. 3a and b), achieving a maximum scavenging ratio of 72.3 ± 2.5% within 5 min at a concentration of 100 mg mL^−1^ (Fig. 3c). Similar trends were observed for DPPH˙ and ABTS˙ radicals, where the hydrogel showed scavenging efficiencies of 78.2 ± 7.6% at 20 mg mL^−1^ within 30 s for DPPH˙ (Fig. 3f) and 54.8 ± 3.2% at 40 mg mL^−1^ within 30 s for ABTS˙ (Fig. 3g). These results indicate that the radical scavenging efficiency of PGDA/CMCS hydrogels improves with increasing hydrogel concentration and exposure time.

The differences in scavenging efficiency between the radicals can be attributed to their distinct chemical properties. PTIO˙, as an oxygen-centered radical with a complex molecular structure, exhibits higher stability and stronger electron-withdrawing properties. Consequently, it requires greater energy or prolonged reaction times for effective reduction. In contrast, DPPH˙ and ABTS˙, both nitrogen-centered radicals, possess relatively lower stability and readily accept electrons, making them more easily reduced under identical conditions.^26^ This explains the faster scavenging ratios observed for DPPH˙ and ABTS˙ radicals compared to PTIO˙.

### 3.4 Photothermal properties of PGDA/CMCS hydrogel

The photothermal effect of PGDA/CMCS hydrogels arises from the spontaneous polymerization of DA into PDA under the presence of oxygen and in a weakly alkaline solution. Due to the catalytic effect of CMCS, the precursor hydrogel solution maintains a mildly alkaline pH (∼9), which facilitates the accelerated polymerization of DA into PDA. The PDA molecules convert absorbed light into heat through non-radiative relaxation of excited electrons, enabling efficient photothermal conversion in the NIR region.

**Fig. 4:**
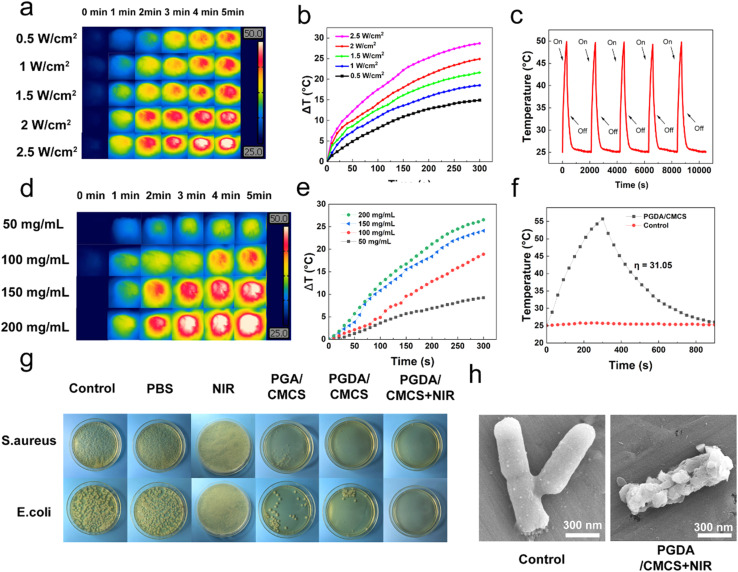
(a) Infrared thermogram of PGDA/CMCS hydrogel at different laser power densities. (b) Photothermal heating curves of PGDA/CMCS hydrogel irradiated with 808 nm laser at different powers. (c) Photothermal cycling test of PGDA/CMCS hydrogel irradiated by 808 nm laser. (d) Infrared thermogram of PGDA/CMCS hydrogel at hydrogel concentration. (e) Photothermal heating curves of PGDA/CMCS hydrogel with different concentrations under 808 nm laser irradiation. (f) Steady-state heating curve of PGDA/CMCS hydrogel. (g) Photographs of *E. coli* and *S. aureus* colonies on LB solid medium after 12 h of incubation. (h) SEM images of *E. coli* of control group and PGDA/CMCS hydrogel after treatment with NIR laser.

To evaluate the photothermal performance of PGDA/CMCS hydrogels, temperature changes were measured under varying NIR laser power densities and irradiation durations. The PGDA/CMCS hydrogel was irradiated with an 808 nm NIR laser at different power densities (0.5, 1.0, 1.5, 2.0, and 2.5 W cm^−2^) for 5 min. At a hydrogel concentration of 150 mg mL^−1^, the thermal imaging results and corresponding temperature curves are shown in Fig. 4a and b, respectively. When irradiated at a power density of 2 W cm^−2^, the hydrogel achieved a temperature increase of 25 °C, which is sufficient to inhibit and partially eliminate the growth of both *E. coli* and *S. aureus*.

PGDA/CMCS hydrogels exhibit excellent photothermal stability, as evidenced by consistent temperature profiles throughout five consecutive on (irradiation) and off (cooling) cycles (Fig. 4c). This indicates that the hydrogels retain their photothermal performance even after repeated use. Additionally, the photothermal conversion capability of PGDA/CMCS hydrogels was evaluated at various concentrations under a fixed laser power density of 2 W cm^−2^. At a concentration of 150 mg mL^−1^, the temperature increase (Δ*T*) exceeded 30 °C (Fig. 4d and e), demonstrating the hydrogel's potential for practical applications.

However, excessively high laser power or hydrogel concentrations can lead to increased energy consumption and material waste. Therefore, for subsequent experiments, a hydrogel concentration of 150 mg mL^−1^ and a laser power density of 2 W cm^−2^ were selected as the optimal conditions. Under these conditions, the photothermal conversion efficiency (*η*) of the PGDA/CMCS hydrogels was calculated to be 31.05% (Fig. 4f), further confirming the hydrogel's efficient photothermal properties.

### 3.5 Antibacterial properties of PGDA/CMCS hydrogel

Severe burn injuries result in the loss of the skin barrier, leading to an increased risk of wound infections, inflammation, delayed healing, and potentially life-threatening complications such as sepsis. Therefore, hydrogels designed for wound healing must possess strong antibacterial properties to mitigate these risks. In this study, the antibacterial efficacy of PGDA/CMCS hydrogels was evaluated against *E. coli* and *S. aureus*, two common pathogens associated with wound infections. Results from bacterial colony analysis after 24 hour co-culture and subsequent agar plating (Fig. 4g) demonstrated that both PGA/CMCS and PGDA/CMCS hydrogels exhibited significant antibacterial activity. This can be attributed to the synergistic effects of CMCS and PGA within the hydrogel matrix. Specifically, CMCS disrupts bacterial membranes through electrostatic interactions, while the acidic environment provided by PGA suppresses bacterial proliferation.

Moreover, the incorporation of NIR irradiation markedly improved the antibacterial efficacy of the PGDA/CMCS hydrogel, while NIR exposure alone demonstrated no appreciable antibacterial activity. Compared to samples without NIR treatment, the bacterial colony counts for both *E. coli* and *S. aureus* were significantly reduced under NIR exposure. This improvement can be explained by the intrinsic photothermal properties of DA, which generated localized hyperthermia upon NIR activation, leading to the disruption of bacterial membrane integrity. SEM imaging provided direct evidence supporting this mechanism (Fig. 4f), as *E. coli* subjected to PGDA/CMCS + NIR treatment displayed ruptured cell surfaces and leakage of intracellular contents, ultimately resulting in bacterial cell death. Such a combination highlights the potential of PGDA/CMCS hydrogels as advanced wound dressings for managing infected wounds, offering both antibacterial efficacy and enhanced wound healing properties. The integration of photothermal activation with conventional antibacterial mechanisms provides a promising strategy for treating complex wound infections.

### 3.6 Biocompatibility properties of PGDA/CMCS hydrogel

**Fig. 5:**
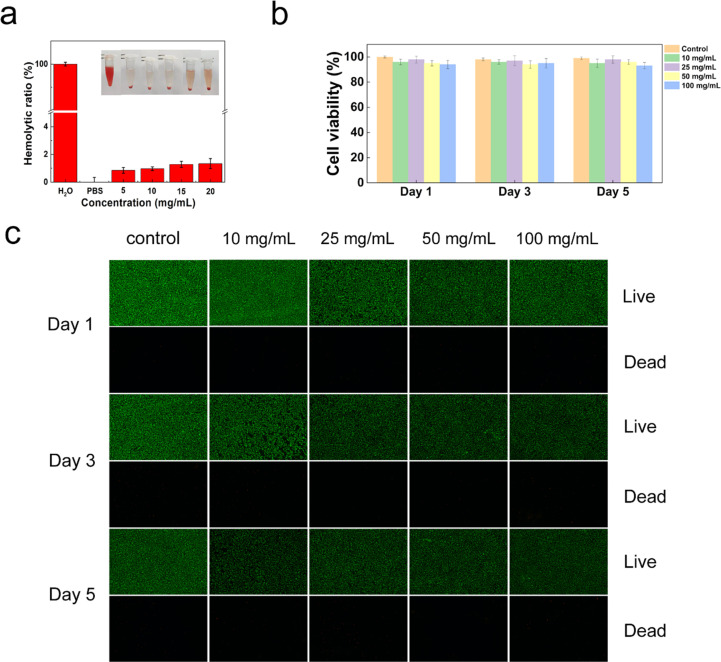
(a) Hemolysis ratios of hydrogel at different concentrations. (b) Cell viability in CCK-8 assays of hydrogel extracts at different concentrations and times. (c) Live/dead staining images of hydrogel at different concentrations and times.

The hemocompatibility of PGDA/CMCS hydrogels was evaluated through a hemolysis assay to assess their potential to induce RBC lysis. Following centrifugation, the mouse RBC solution treated with water exhibited a bright red color, indicating complete hemolysis due to RBC rupture. In contrast, when co-cultured with PGDA/CMCS hydrogel extracts at concentrations of 0, 5, 10, 15 and 20 mg mL^−1^, the *in vitro* hemolysis ratio remained below 5% (Fig. 5a), meeting the threshold of acceptable hemocompatibility set by international standards (<5%). These findings align with previous reports that have demonstrated hydrogels with carboxymethyl CMCS or PGA components often show excellent blood compatibility due to their biocompatible and non-immunogenic nature.^27^

Cytocompatibility was further examined by incubating PGDA/CMCS hydrogels with L929 fibroblast cells for 1, 3, and 5 days, followed by a CCK-8 assay to evaluate cell viability. Across all tested hydrogel extract concentrations (10, 25, 50, and 100 mg mL^−1^), cell viability consistently exceeded 90% (Fig. 5b), indicating negligible cytotoxic effects. Live/dead staining further confirmed this observation (Fig. 5c); no significant differences were observed in L929 fibroblasts cultured with different concentrations of hydrogel extracts, compared to the control group. Additionally, cell density increased over time, reflecting normal cell proliferation and indicating that the hydrogel microenvironment supports cellular growth without inhibitory effects.

### 3.7 Hemostatic properties of PGDA/CMCS hydrogel

**Fig. 6:**
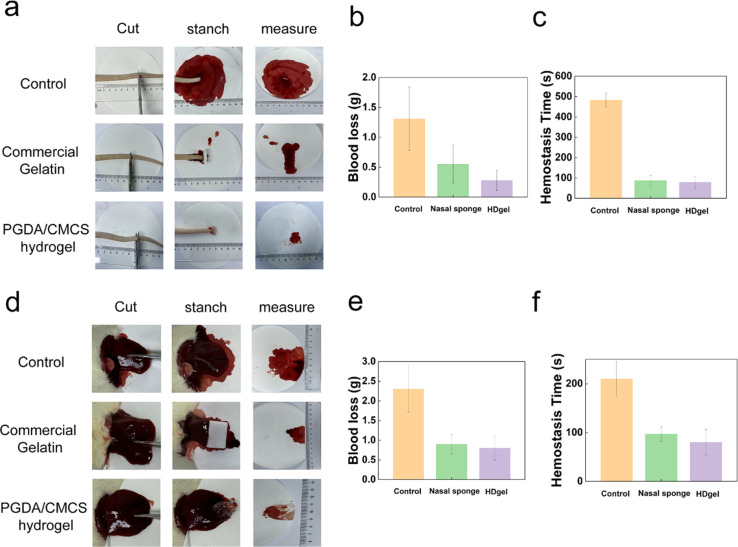
(a) Hemostasis images of SD rat tail amputation. (b) Blood loss during tail amputation hemostasis experiment. (c) Hemostasis time during tail amputation. (d) Hemostasis images of SD rat liver. (e) Blood loss during liver hemostasis experiment. (f) Hemostasis time during liver hemostasis.

The hemostatic performance of PGDA/CMCS hydrogels was evaluated using SD rat tail amputation and liver hemorrhage models, which are widely recognized as reliable methods for assessing the hemostatic efficacy of biomaterials. In the SD rat tail amputation model, the application of PGDA/CMCS hydrogels to the wound site (Fig. 6a) successfully achieved hemostasis within 60 s. No further bleeding was observed after hemostasis, and absorbent paper remained clean throughout the observation period. Quantitative analysis revealed that, compared to the control group, the PGDA/CMCS hydrogel group exhibited less total blood loss (Fig. 6b). Furthermore, comparative analysis of blood loss and hemostasis time (Fig. 6c) demonstrated that PGDA/CMCS hydrogels outperformed commercially available hemostatic gelatin, exhibiting faster clotting and reduced blood loss, thus indicating superior hemostatic efficiency.^28^

Similarly, in the liver hemorrhage model, pre-prepared PGDA/CMCS hydrogels were applied to the liver wound site (Fig. 6d). Gentle compression of the hydrogel against the wound surface resulted in effective hemostasis within 1 min. Compared with the control group and commercial hemostatic gelatin, the PGDA/CMCS hydrogel group exhibited a significant reduction in blood loss during the procedure. Post-hemostasis observations confirmed that absorbent paper remained clean, and no further bleeding occurred in the PGDA/CMCS hydrogel group. Comparative analysis of total blood loss (Fig. 6e) and hemostasis time (Fig. 6f) further substantiated the superior hemostatic performance of PGDA/CMCS hydrogels, which significantly reduced blood loss and shortened hemostasis time compared to other commercial products, further confirming the practical advantages of the proposed material.^29^

These findings demonstrate the potential of PGDA/CMCS hydrogels as an effective hemostatic material. The synergy between PGDA and CMCS enables rapid hemostasis, while dopamine provides strong adhesion, forming a physical barrier and promoting platelet aggregation to initiate coagulation. Its catechol groups can interact with blood proteins and cell membranes, facilitating clot formation and stabilizing the clot structure.^30^ Moreover, CMCS facilitates adhesion to the wound surface through electrostatic interactions, promoting clot formation, while the inherent hydrophilicity of the hydrogel matrix accelerates water absorption from the blood, thereby enhancing the concentration of coagulation factors at the wound site.^31^ Furthermore, the mechanical stability of the hydrogel ensures firm coverage of the wound surface, reducing the likelihood of recurrent bleeding.

### 3.8 Wound healing in severe burn model of PGDA/CMCS hydrogels

**Fig. 7:**
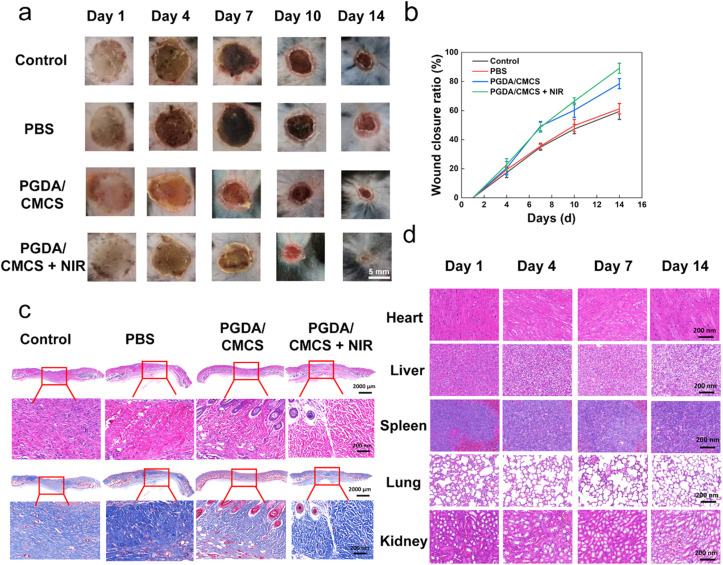
(a) Wound healing images of C57 mice treated with different components over 14 days. (b) Wound healing rate of C57 mice with different components over 14 days. (c) H&E and Masson's trichrome staining of wound granulation tissue sections from different groups on day 14. (d) H&E staining of heart, liver, spleen, lung, and kidney tissue sections from PGDA/CMCS-treated groups at different time points.

Creating a moist environment and preventing bacterial infection are crucial for effective burn wound healing. The treatment of deep second-degree burns in C57 mice demonstrated that wound healing ratios in all experimental groups were faster than those in the control group. Photographs of the wounds (Fig. 7a) and statistical analysis of wound closure ratios (Fig. 7b) revealed that the PGDA/CMCS group and PGDA/CMCS + NIR group achieved significantly faster healing compared to the PBS group and control group. Notably, the PGDA/CMCS + NIR group exhibited the highest wound healing ratios among all groups, suggesting that the antibacterial properties of the PGDA/CMCS hydrogel, combined with NIR photothermal therapy, effectively prevented bacterial infections and accelerated wound closure.

The incorporation of dopamine (DA) into the PGDA/CMCS hydrogel contributed to its superior antioxidant properties and photothermal performance, further promoting wound healing. Dopamine's antioxidant activity effectively neutralized reactive oxygen species (ROS), reducing oxidative stress in the wound microenvironment, which is known to impede the wound healing process. Simultaneously, the photothermal effect of PGDA under NIR irradiation elevated local temperatures, which not only enhanced antibacterial efficacy but also stimulated blood circulation and cellular activity in the wound bed. Importantly, when the combined effects of antibacterial, antioxidant, and photothermal therapies were employed, the wound healing ratio significantly exceeded that of using these therapies individually.

Histological analyses further supported these findings. By day 14, the PGDA/CMCS + NIR group exhibited fewer inflammatory cells in the wound bed and fewer unhealed wounds, indicating that photothermal therapy in the early stages of treatment effectively accelerated the healing process (Fig. 7c). Epithelialization was observed in all groups, but distinct differences were noted. The blank and PBS groups were still transitioning through the epithelial regeneration phase, while the PGDA/CMCS and PGDA/CMCS + NIR hydrogel-treated groups had progressed to the functional skin reconstruction stage, characterized by significantly thinner epithelium and enhanced re-epithelialization. Furthermore, H&E staining of vital organs (heart, liver, spleen, lungs, and kidneys) at various time points (days 1, 4, 7 and 14) revealed no signs of inflammation, demonstrating the biosafety of PGDA/CMCS hydrogels (Fig. 7d). Collectively, these results confirm the therapeutic potential of PGDA/CMCS hydrogels for the treatment of burn wounds, particularly when combined with NIR photothermal therapy. This approach not only accelerates wound healing but also minimizes infection and inflammation, offering a safe and effective strategy for managing burn injuries.

## 4. Conclusion

This study synthesized a PGDA/CMCS hydrogel using PGA, DA, and CMCS as burn wound dressings. The PGDA/CMCS hydrogel precursor solution can be injected into burn wounds. Upon exposure to EDC/NHS, the hydrogel exhibited strong bioadhesion, shape adaptability, and water retention capacity, allowing it to conform to irregular wound shapes and maintain a moist environment conducive to healing. The hydrogel showed good biocompatibility and antibacterial properties, effectively preventing wound infection. Additionally, it demonstrated excellent free radical scavenging ability, enhancing burn wound repair, and promoting the regeneration of intact skin structures, indicating significant potential for clinical applications.

## Author contributions

CW: conceptualization; data curation; formal analysis; investigation; methodology; software; writing – original draft; HJ: conceptualization; data curation; formal analysis; methodology; QZ: conceptualization; data curation; formal analysis; YJ: conceptualization; data curation; formal analysis; YB: conceptualization; software; funding acquisition; SF: software; JY: resources; writing – original draft; HW and JX: conceptualization; data curation; formal analysis; funding acquisition; investigation; methodology; project administration; resources; software; supervision; validation; visualization; writing – original draft; writing – review & editing.

## Conflicts of interest

The authors declare that they have no known competing financial interests or personal relationships that could have appeared to influence the work reported in this paper.

## Supplementary Material

RA-015-D5RA03968A-s001

## Data Availability

Data will be made available on request.
