# Supplementary material for: A multifunctional hydrogel with hemostatic and photothermal properties for burn repair
Source: RSC Adv. 2025 Jul 14;15(30):24497–509. doi: 10.1039/d5ra03968a (PMC12257075; doi:10.1039/d5ra03968a)
Supplement: RA-015-D5RA03968A-s001 [file RA-015-D5RA03968A-s001.pdf]

Supplementary Information (SI) for RSC Advances.
This journal is © The Royal Society of Chemistry 2025

Supporting Information

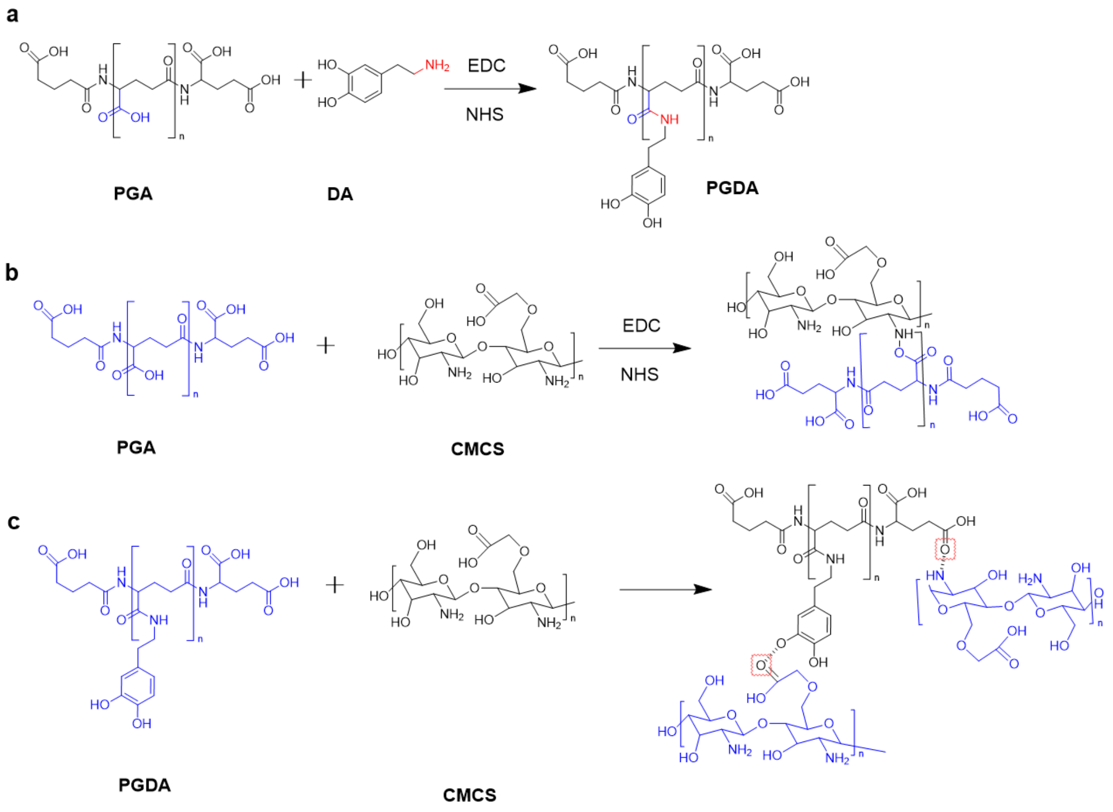

Figure S1. Schematic illustration of the chemical interactions involved in PGDA/CMCS hydrogel formation. (a) Covalent grafting of DA onto PGA via EDC/NHS-mediated amide bond formation. (b) Unreacted carboxyl groups on PGDA form Schiff base linkages with the amino groups on CMCS, contributing to covalent network formation. (c) Hydrogen bonding interactions between the catechol groups of PGDA and carboxyl groups of CMCS further enhance the hydrogel network.

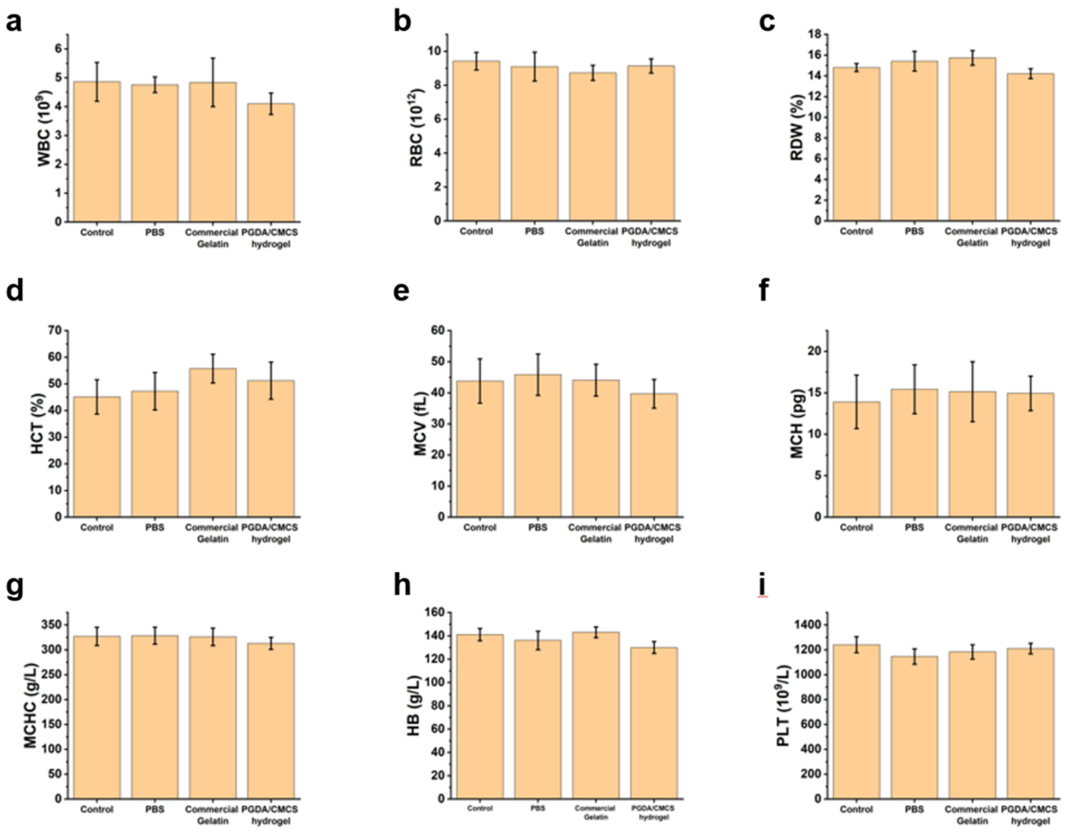

Figure S2. Blood routine test results of different groups on day 14. (a) hemoglobin; (b) hematocrit; (c) mean corpuscular hemoglobin; (d) mean corpuscular hemoglobin concentration; (e) mean corpuscular volume; (f) platelet count; (g) red blood cell count; (h) red blood cell volume distribution width; (i) White blood cell count

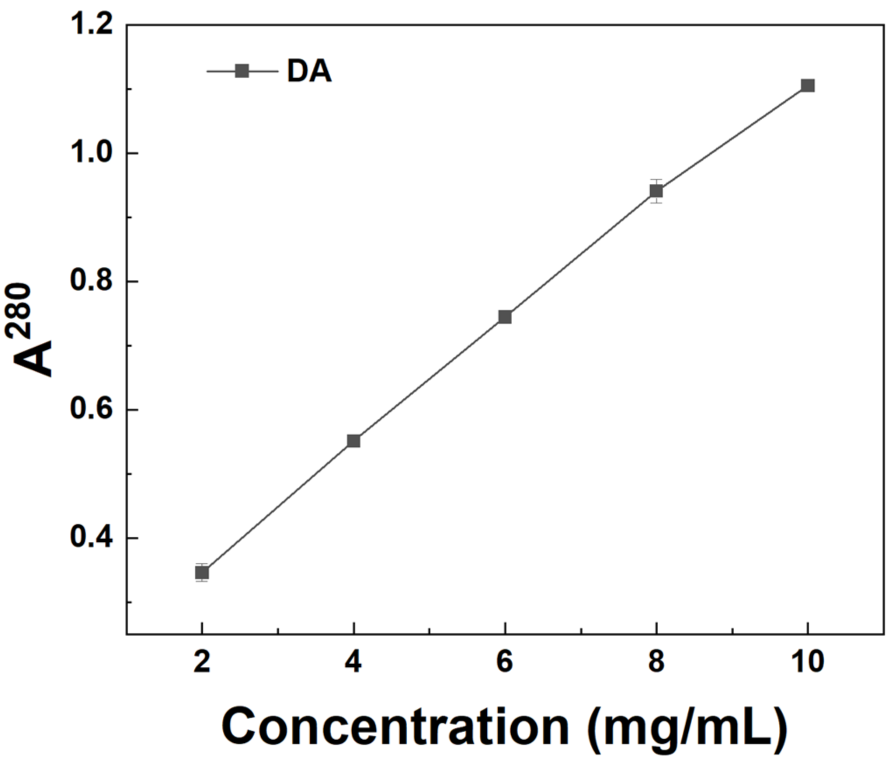

Figure S3. Standard calibration curve of DA at 280 nm. The absorbance values were measured for DA solutions with concentrations ranging from 2 to 10 mg/mL. A linear relationship was observed between the concentration and absorbance.

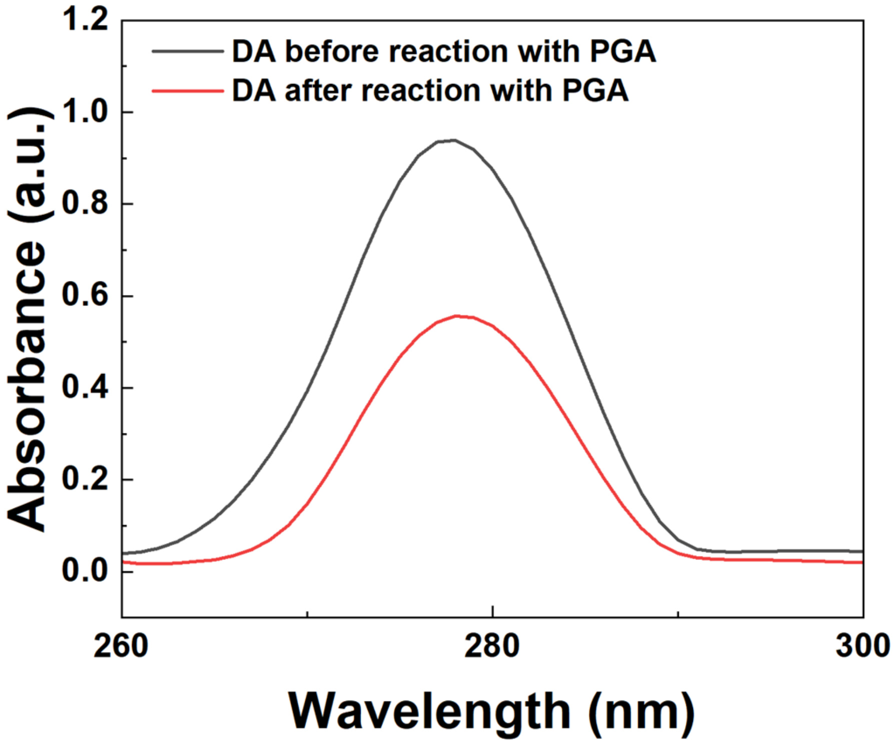

Figure S4. UV－vis absorption spectra of DA before and after reaction with PGA. A significant decrease in the absorbance peak at ~280 nm after reaction indicates the successful grafting of DA onto PGA.

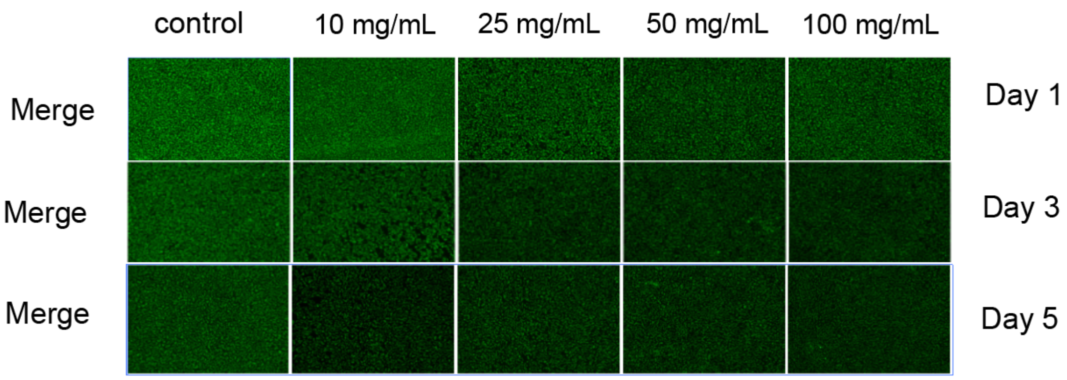

Figure S5. Live/dead staining merged fluorescence images of hydrogel samples at various concentrations and incubation times. Green fluorescence indicates live cells (Calcein-AM), while red fluorescence indicates dead cells (PI). The overlay images demonstrate the cytocompatibility of the hydrogels under different conditions.
